# Computational Identification of Most Deleterious Missense Mutations in Human PD-1 Gene

**DOI:** 10.1155/2023/4360203

**Published:** 2023-08-07

**Authors:** Haitham Ahmed Al-Madhagi

**Affiliations:** Thamar University, Dhamar, Yemen

## Abstract

Traditional cancer treatment approaches are often hindered by the presence of toxic side effects and the high rate of relapse observed in treated organs. In contrast, novel immunotherapeutic strategies targeting immune checkpoint inhibitors, particularly PD-1, have demonstrated promising results with minimal adverse effects. However, the emergence of immunotherapeutic-resistant tumors, predominantly caused by intrinsic mutations, poses a significant obstacle to successful treatment outcomes. Consequently, the primary objective of this study was to screen for the most detrimental missense mutations in the PD-1 gene associated with immunotherapeutic resistance. To achieve this aim, a comprehensive screening process utilizing 20 web servers, incorporating both sequence- and structure-based methodologies, was undertaken. Through meticulous analysis and mutual disease association sorting, four specific missense mutations were successfully identified. These mutations, namely, R38C, D61V, R94C, and D117V, emerged as the leading contributors to genetic cancer progression and immunotherapeutic resistance against PD-1 blockers. The findings presented in this study are supported by multiple lines of evidence. A thorough examination of protein topology, structural alignment, docking interactions with PD-L1, and protein flexibility collectively confirmed the pathogenic nature of these sorted mutations. By considering these various aspects, we have gained a comprehensive understanding of the underlying mechanisms driving immunotherapeutic resistance. In conclusion, the comprehensive screening process undertaken in this study has successfully identified R38C, D61V, R94C, and D117V as the primary mutations contributing to genetic cancer progression and immunotherapeutic resistance against PD-1 blockers. The integration of protein topology analysis, structural alignment, docking studies with PD-L1, and assessment of protein flexibility have collectively provided robust evidence to support the pathogenic significance of these mutations.

## 1. Introduction

Cancer, a devastating disease affecting a substantial number of individuals worldwide, remains a significant global health concern. With an annual incidence of approximately 2 million cases, it tragically accounts for an estimated 608,000 deaths each year, and this number is projected to escalate to 22 million deaths by 2030 [1]. The current therapeutic approaches available for cancer treatment encompass three primary modalities: surgery, radiotherapy, and chemotherapy. The selection of treatment options depends on the type and stage of cancer in question. Typically, surgical intervention is employed as the initial step, followed by a combination of radiotherapy and chemotherapy. This comprehensive approach aims to reduce the size of cancerous tissue through surgery and radiotherapy while utilizing chemotherapy for long-term administration to impede the proliferation of cancer cells [2]. Despite the relative efficacy of these treatment strategies, no definitive cure has been achieved. Moreover, the high cost and considerable toxicity associated with chemotherapy necessitate a pressing need for the exploration of novel therapeutic avenues [3].

The modern arena of chemotherapy is immune-based therapeutics (immunotherapy). The immune system is requested to regularly perform surveillance to detect and kill cells transforming into cancerous. While it is substantially important for immune cells to first identify the cells undergoing cancer transformation, impedance is to alleviate or overcome immunosuppression imposed by cancer tissue preventing the identification step [4]. Immune checkpoint inhibitors (ICIs) revolutionized the field of cancer immunotherapy by overcoming immunosuppression. Two major ICI targets include cytotoxic T-lymphocyte-associated antigen 4 (CTLA-4) and programmed cell death 1 (PD-1). CTLA-4 is upregulated in regulatory T cells resulting in downregulation of the immune response toward cancer cells. On the other hand, PD-1 upon binding to its inhibitory ligand (PD-L1) is prevented from causing apoptosis to infected host cells as well as cancer cells, keeping cancer progression [5]. ICI treatment has been shown to satisfy the desired therapeutic index with minimal side effects in clinical trials [6].

However, the efficacy of immune checkpoint inhibitor (ICI) immunotherapy has been hindered by the emergence of resistance mechanisms in cancer cells. These resistant cancerous cells have the ability to counteract the effects of ICI therapy by acquiring multiple mutations in the targets of ICIs and their corresponding natural ligands. This resistance poses a significant challenge, as the cancer cells can display aggressive behavior towards immune cells in advanced stages. Notably, the relapse rate after immunotherapy is estimated to be as high as 1 in every 3 melanoma patients [7].

Herein, the aim of the present theoretical work is to sort and shortlist the most pathogenic missense mutations of PD-1 through several sequence-based and structure-based analysis methods and to assess the structural and binding effects of such mutations.

## 2. Methodology

**Figure 1:**
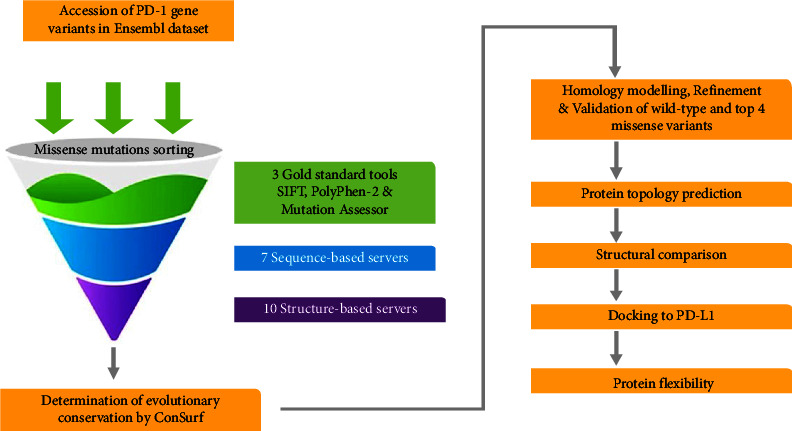
Flowchart elucidating the workflow of the present study.

The strategy employed in the present theoretical work is summarized in Figure 1. However, the detailed explanation of each step is given.

### 2.1. Sequences Accession

The sequence of PD-1 was obtained from Ensembl dataset [8] (Ensembl ID: ENSG00000188389). The variant table of the same dataset was utilized for retrieving and calculating the total and classification of mutations.

### 2.2. Sorting Mutations

Only missense mutations were considered for further analysis. The retrieved missense mutations were subjected to 3 stages of sorting. The first stage involved ruling out the medium and less pathogenic mutants via the built-in tools, namely, SIFT [9], PolyPhen-2 [10], and MutaionAssessor [11]. Stage two involves sequence-based prediction via SNAP2 [12], MutPred2 [13], SNP&GO [14], SuSPect [15], PANTHER [16], PMut [17], and DEOGEN 2 [18] tools, whereas the third stage represented structure-based predictions using MUpro [19], I-Mutant 2 [20], iStable 2 [21], CUPSAT [22], SDM [23], mCSM [24], DUET [25], MAESTROweb [26], DynaMut 2 [27], and DeepDDG [28] servers (20 in total). This ensures the obtaining of consensus disease-associated missense mutations and, at the same time, excluding mutations with less probability of neutral variants.

### 2.3. Determination of Evolutionary Conservation

The consensus mutations were subjected to evolutionary conservation exploration to test the position and functional impact imposed by those missense variants. This was accomplished utilizing the ConSurf tool [29].

### 2.4. Homology Modelling, Refinement, and Validation

After ruling out the less pathogenic missense mutations and validating the most deleterious ones, the 4 most risky mutations, together with the native protein (Uniprot ID# Q15116), were inputted to the SWISS-Model [30] for homology modelling the tertiary structure and consecutive refinement through the GalaxyRefine web portal [31]. The built model for wild-type PD-1 was checked using the Ramachandran plot calculated using UCSF Chimera v1.16 [32] and further by the ProsA program [33].

### 2.5. Prediction of Protein Topology

Utilizing the UniProt ID of PD-1 (Q15116), protein topology and its invagination into the cell membrane were evaluated using the TOPCONS server [34] and validated and depicted using Protter [35]. Those servers also reveal the signal peptide, posttranslational modifications, and disulfide bonds.

### 2.6. Structural Comparison

To determine the effect of SNPs on the tertiary structure of PD-1 wild-type and its variants, structural superimposition was performed using RCSB pairwise structure alignment [36]. Both rigid and flexible modes of superimposition were tested.

### 2.7. Docking to PD-L1

In order to give crucial results regarding the effect of missense variants on the function of PD-1, protein docking was performed via the HDock platform [37] to evaluate wild-type PD-1 and its variants binding to the native inhibitory protein PD-L1 (PDB ID# 5IUS).

### 2.8. Protein Flexibility

The flexibility of all variants was examined using the CABS-Flex 2 server (http://biocomp.chem.uw.edu.pl/CABSflex2/index) [38]. The number of cycles and cycles between trajectory frames was set at 50; otherwise, the remaining parameters were set as default. Root mean square fluctuation (RMSF) data of all variants were plotted using Microsoft® Excel 2019.

## 3. Results and Discussion

**Figure 2:**
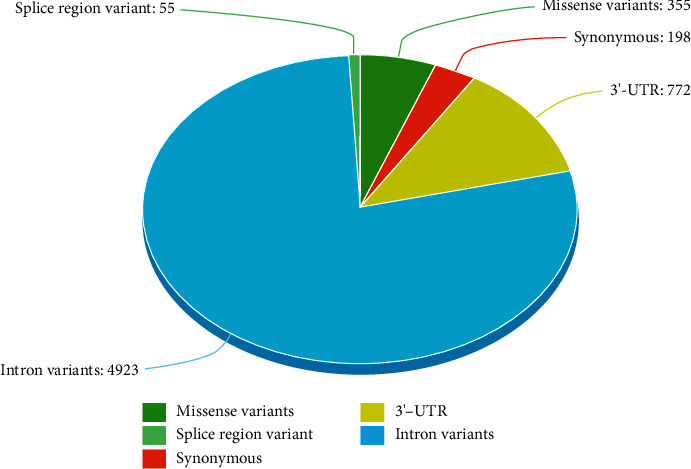
Distribution frequency of mutations in the PD-1 gene.

There were more than 6,300 mutations found in the Ensembl dataset concerning the PD-1 gene. Among these, 355 missense variants were observed, and the majority were intronic mutations as elucidated in Figure 2.

### 3.1. Sequence-Based Predictions

The 3 gold standard tools, namely, SIFT, PolyPhen-2, and MutationAssessor, were used for ruling out the neutral missense variants from damaging ones. The SIFT algorithm listed 100 SNPs as deleterious. On the other hand, PolyPhen-2 classified 49 missense variants as probably damaging, whereas only 4 variants demonstrated high impact in MutationAssessor. Therefore, upon comparison of the three tools, four deleterious missense SNPs were found in common (summarized in Table 1).

**Figure 3:**
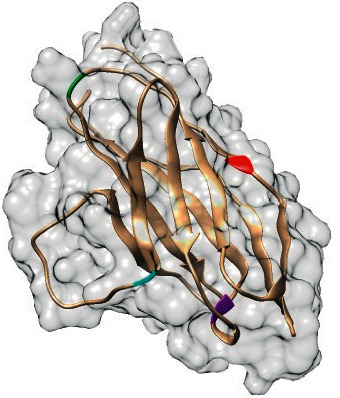
3D structure of native PD-1 along with the predicted hotspot positions of consensus mutations. Positions 38, 61, 94, and 117 are shown in red, green, cyan, and purple, respectively.

The positions of those consensus mutations are shown in Figure 3. Subsequently, the top 4 deleterious mutations were evaluated through 7 sequence-based predictions, which are summarized in Table 2.

Among the utilized tools, only the DEOGEN web server classified the four variants as deleterious. The remaining tools exhibited fluctuating findings; i.e., SNAP2 sorted 3 as effect, whereas the rest tools classified ≤2 variants as damaging.

### 3.2. Structure-Based Predictions

Similar to sequence-based servers, 2 servers (MUpro and DeepDDG) classified the effect of mutation on stability as “destabilizing” followed by MAESTROweb (3 destabilizing mutations), while the rest of the tools predicted two variants, in common, as destabilizing mutations (Table 3).

### 3.3. Determination of Evolutionary Conservation

**Figure 4:**
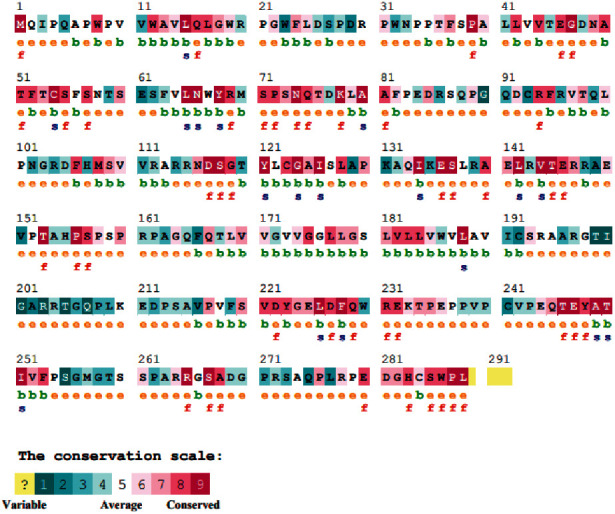
Evolutionary conservation of the individual residues along with the corresponding surface accessibility. b: buried; e: exposed; f: functional; s: structural.

As illustrated in Figure 4, the four hotspot mutation sites, namely, 38, 61, 94, and 117, were exposed on the surface. In addition, the obtained data suggest the implication of 2 missense mutations (R94C and D117V) in functional roles. This confirms the deleterious effects of substituting such highly conserved residues. The other sites, 38 and 61, were variable and thus imposed less effect on the protein function. Accordingly, the most pathogenic missense variants are R94C and D117V.

### 3.4. Homology Modelling

**Figure 5:**
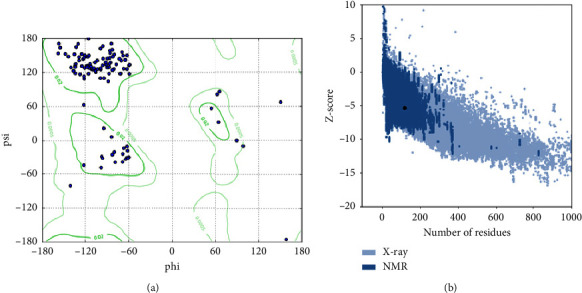
Validation of the generated and refined model of PD-1 via the Ramachandran plot (a) and ProsA (b).

The generated model had 95.1% Rama favoured, 4.9% residues in the allowed regions with no poor rotamers (Figure 5(a)). Validation via the ProsA server produced a *Z*-score of −5.31. Furthermore, the black circle lies within the NMR revealed structures, mirroring the high quality of the generated model (Figure 5(b)).

### 3.5. Protein Topology

**Figure 6:**
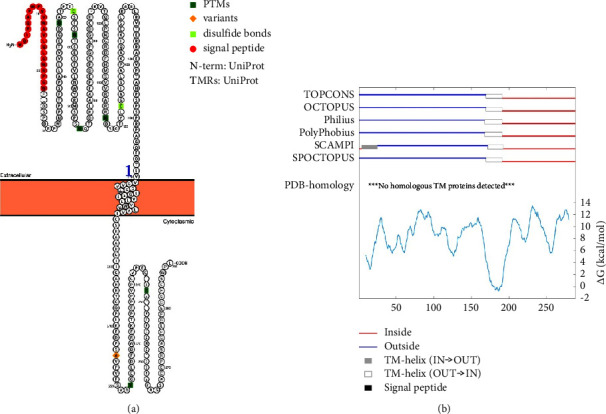
Obtained protein topology as predicted by Protter (a) and TOPCONS (b).

Only a small portion of the total protein was predicted to span the membrane (residues 170–190). Residues before 170 constitute the extracellular domain, whereas the residues after 190 form the intracellular domain. The two examined servers are in a highly significant agreement with respect to the transmembrane stretch (Figure 6). Therefore, all tested variants lie within the extracellular domain, in which the ligand-binding pocket is located.

### 3.6. Structural Comparison

**Figure 7:**
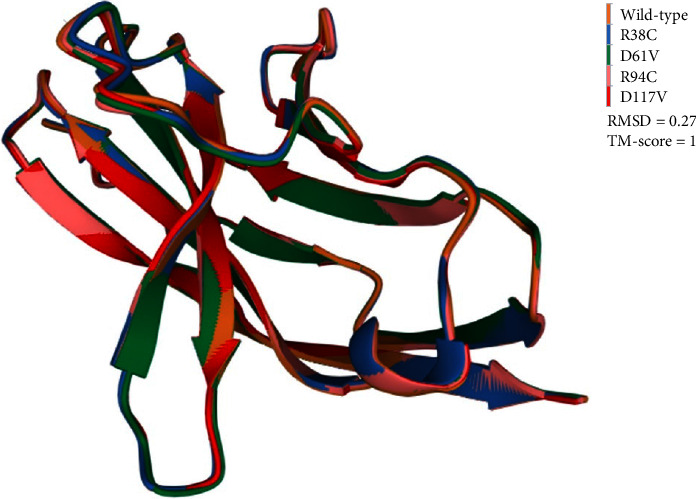
Structural superimposition of the wild-type PD-1 together with the top four missense variants. RMSD and TM-scores are also shown.

**Figure 8:**
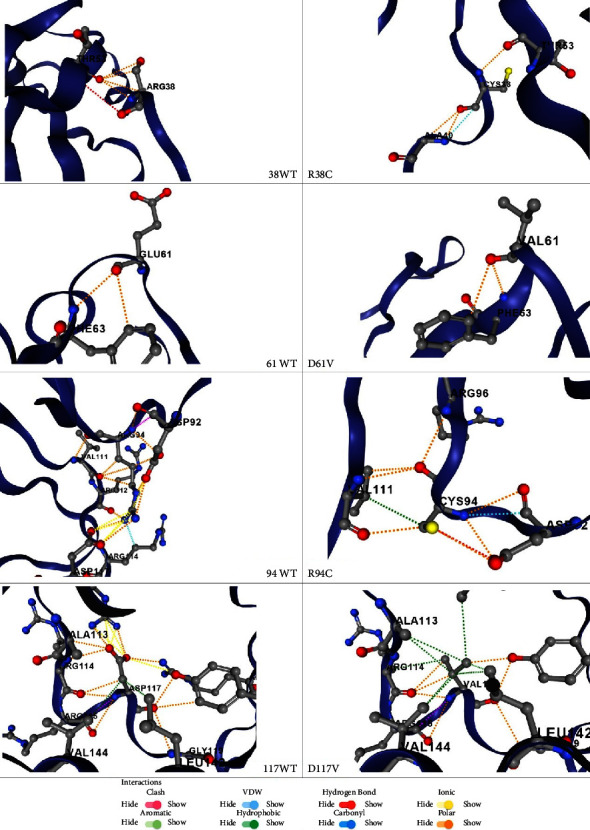
Individual structural consequences of the four variants predicted using the DynaMut 2 server.

To see the effect of SNPs on the tertiary structure of PD-1 variants, structural superimposition was performed using RCSB pairwise structure alignment. The rigid as well as flexible aligned variants exhibited a root mean square deviation (RMSD) of 0.27 and TM-score of 1. This indicates that the SNPs have very weak impact on the whole protein structure, albeit the variants were distributed from residue 38 to 117 (Figure 7). Nevertheless, an in-depth analysis of individual variants using the DynaMut 2 server revealed steric hindrance (intermolecular clashes) and repulsive interactions predominantly in the D117V variant since the first and third variants had stabilizing impact on the whole protein architecture. The individual structural consequences of the variants are depicted in Figure 8.

### 3.7. Docking to PD-L1

In order to give crucial results regarding the effect of missense variants on the function of PD-1, protein docking was performed via the HDock platform to evaluate wild-type PD-1 and its variants binding to the native inhibitory protein PD-L1 (PDB ID# 5IUS).

As shown in Table 4, the wild-type protein exhibited relatively weaker binding to its native inhibitory protein PD-L1. In contrast, the four variants showed stronger binding energy to PD-L1. This demonstrates the deleterious effect of missense mutations on protein affinity since stronger binding means, in this case, inability of the PD-1 protein to avoid PD-L1 binding, and thus, no apoptosis takes place in malignant cells.

### 3.8. Protein Flexibility

**Figure 9:**
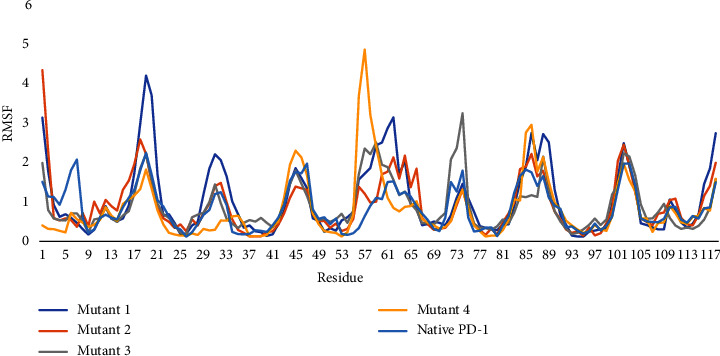
RMSF of all variants compared with native PD-1 protein.

The RMSF diagram showed marked fluctuations in all variants at different positions. Nevertheless, there were relatively consensus peaks at certain positions, namely, 17–21, 28–35, 42–48, 55–65, 72–76, 83–92, and 99–105 (Figure 9). RMSF data indicate the changed dynamics of the variants in comparison with the native protein. Hence, RMSF data emphasize the deleterious effect of all variants.

## 4. Discussion

Conventional chemotherapy approaches exert their therapeutic effects by interfering with various cellular processes such as the cell cycle, DNA replication, cell metabolism, or microtubule assembly, ultimately impeding tumor growth [39]. However, certain cytotoxic drugs, including oxaliplatin and anthracycline, not only induce immunogenic cell death but also trigger an antitumor immune response [40]. These drugs not only enhance the activity of immune effector cells but also modulate the tumor microenvironment by eliminating and recruiting tumor-infiltrating macrophages and suppressing suppressor T cells. When combined with immune checkpoint inhibitors (ICIs), particularly those targeting the PD-1/PD-L1 axis, they hold great promise for the treatment of various cancers, such as non-small cell lung carcinoma [41]. It is important to note that neither conventional chemotherapy nor immunotherapy alone is sufficient to address the complexity and dynamic nature of cancer, as evidenced by the large number of mutations observed. These mutations can confer resistance to treatment or lead to drug tolerance [42]. Therefore, the objective of the present theoretical work was to analyze the most prevalent deleterious missense mutations in the PD-1 gene utilizing various bioinformatics methods.

Upon selection of the golden standard tools, SIFT, PolyPhen-2, and MutationAssessor, 4 consensus missense variants were obtained. Unfortunately, the application of multiple tools, whether sequence-based or structure-based, failed to confirm the findings of the preliminary assortment. Only DEOGEN and MUpro showed identical results, whereas other tools used resulted in conflicting output. From an evolutionary perspective, R94C and D117V are in the highly conserved hotspot region and adopt functional roles in the protein as predicted by using the ConSurf web server. Moreover, from the protein topology results, it can be inferred that all four variants lie within the extracellular domain at the N-terminal, where the cognate ligands bind [43]. This confirms the deleterious effects of the shortlisted variants. The structural comparison among the found missense variants along with the wild-type protein had no impact on the whole protein fold as the TM-score was predicted to be 1, indicating almost complete typical alignment. However, docking to PD-L1 via the HDock platform demonstrated strong binding of the four variants compared to control which reflects tumor-promoting and antiapoptotic actions. It should be noted that DeepDDG results were substantially compatible with protein docking output with the notion that all the four variants decreased PD-1 stability in addition to sorting R94C and D117V as the most dangerous ones. Once bound to PD-L1, PD-1 plays a critical role in dampening the immune response and inducing self-tolerance through interfering with external apoptotic signaling cascade initiation particularly in tumor cells [44]. Protein fluctuations expressed as RMSF of all variants emphasized the destabilizing impact of the examined missense mutations. Overall, the cumulative demonstrations in the current study confirm the deleterious effect of R38C, D61V, R94C, and D117V on the PD-1 protein structure and binding affinity accounting for the impact of mutation on cancer progression and consecutively on resistance to drugs.

## 5. Conclusion

The objective of this study was to identify the most pathogenic missense variants in the PD-1 gene through the utilization of three widely recognized tools: SIFT, PolyPhen-2, and MutationAssessor. As a result, four highly deleterious mutations were identified and further investigated through sequence-based predictions and subsequently through structure-based predictions using a comprehensive set of 17 servers. Notably, the analysis conducted with the ConSurf tool revealed that two of these mutations (R94C and D117V) occurred in highly conserved regions, indicating their potential damaging effects. In addition, structural superimposition demonstrated minimal differences in the overall protein structure among the variants. The subsequent step involved docking simulations against the natural ligand PD-L1, which further confirmed the pathogenic nature of the mutations R38C, D61V, R94C, and D117V. Furthermore, the assessment of protein flexibility corroborated the significance of these mutations in cancer progression and the recurrence of tumors following immunotherapy treatment. It is worth noting that for assessing the stability effects of mutations on proteins, the DeepDDG web portal is recommended as a preferable tool. These findings shed light on the detrimental impact of specific missense mutations in the PD-1 gene, providing valuable insights into their role in cancer progression and resistance to immunotherapy. Future research may benefit from employing the DeepDDG web portal to further evaluate the stability effects of these mutations on the protein structure and function.

## Figures and Tables

**Table 1:** Shortlisting of the most probable disease-associated missense variants.

Tools	R38C	D61V	R94C	D117V
SIFT	0 (Deleterious)	0 (Deleterious)	0 (Deleterious)	0 (Deleterious)
PolyPhen-2	0.999 (Probably damaging)	0.998 (Probably damaging)	1 (Probably damaging)	1 (Probably damaging)
MutationAssessor	0.953 (High)	0.955 (High)	0.953 (High)	0.955 (High)

**Table 2:** Sequence-based predictions of the top 4 missense variants.

Tools	R38C	D61V	R94C	D117V
SNAP2	−14 (Neutral)	26 (**Effect**)	71 (**Effect**)	83 (**Effect**)
MutPred 2	0.109 (Neutral)	0.259 (Neutral)	0.768 (**Pathogenic**)	0.805 (**Pathogenic**)
SNP&GO	7 (Neutral)	3 (Neutral)	3 (Neutral)	3 (**Disease**)
SuSPect	69 (**Disease-associated**)	15 (Neutral)	91 (**Disease-associated**)	12 (Neutral)
PANTHER	0.27 (Probably benign)	0.27 (Probably benign)	0.27 (Probably benign)	0.5 (**Possibly damaging**)
PMut	0.07 (Neutral)	0.11 (Neutral)	0.45 (Neutral)	0.69 (**Disease**)
DEOGEN	0.85 (**Deleterious**)	0.64 (**Deleterious**)	0.87 (**Deleterious**)	0.89 (**Deleterious**)

**Numbers** indicate the probable mutation effect score, **while bold** indicates deleterious mutations.

**Table 3:** Structure-based predictions of the top 4 missense variants.

Tools	R38C	D61V	R94C	D117V
MUpro	−0.187 (**Decrease stability**)	−0.326 (**Decrease stability**)	−1.307 (**Decrease stability**)	−0.734 (**Decrease stability**)
I-Mutant 2	2 (**Decrease**)	1 (Increase)	5 (**Decrease**)	5 (**Decrease**)
iStable 2	−0.494 (**Decrease**)	0.507 (Increase)	−0.90 (**Decrease**)	−1.24 (**Decrease**)
CUPSAT	−1.4 (**Destabilizing**)	−1.94 (**Destabilizing**)	0.29 (Stabilizing)	0.53 (Stabilizing)
SDM	1.32 kcal/mol (Stabilizing)	−0.17 kcal/mol (**Destabilizing**)	−0.18 kcal/mol (**Destabilizing**)	1.13 kcal/mol (Stabilizing)
mCSM	−0.43 kcal/mol (**Destabilizing**)	0.14 kcal/mol (Stabilizing)	−1.033 kcal/mol (**Destabilizing**)	1.009 kcal/mol (Stabilizing)
DUET	0.162 kcal/mol (Stabilizing)	0.347 kcal/mol (Stabilizing)	−0.97 kcal/mol (**Destabilizing**)	1.492 kcal/mol (Stabilizing)
MAESTROweb	0.51 kcal/mol (**Destabilizing**)	1.428 kcal/mol (**Destabilizing**)	0.88 kcal/mol (**Destabilizing**)	−0.374 kcal/mol (Stabilizing)
DynaMut 2	0.23 kcal/mol (Stabilizing)	−0.7 kcal/mol (**Destabilizing**)	1.51 kcal/mol (Stabilizing)	−0.54 kcal/mol (**Destabilizing**)
DeepDDG	−0.279 kcal/mol (**Destabilizing**)	−0.160 kcal/mol (**Destabilizing**)	−2.146 kcal/mol (**Destabilizing**)	−2.833 kcal/mol (**Destabilizing**)

**Numbers** indicate the probable mutation effect score, **while bold** indicates deleterious mutations.

**Table 4:** Protein docking profile between the wild-type PD-1 and the top 4 missense variants by HDock.

Receptor	Wild-type	R38C	D61V	R94C	D117V
Docking score (kcal/mol)	−224.08	−259.43	−237.61	−238.76	−237.32

## Data Availability

All the obtained data are included within the article.
